# Professional Factors Associated with Case Resolution without Referrals of Orofacial Pain Cases to Secondary Dental Care by Telehealth in Brazil: A Cross-Sectional Study in 2019 and 2020

**DOI:** 10.3390/life13010029

**Published:** 2022-12-22

**Authors:** Ricardo Luiz de Barreto Aranha, Renata de Castro Martins, Ligia Cristelli Paixão, Mauro Henrique Nogueira Guimarães de Abreu

**Affiliations:** 1 School of Dentistry, Universidade Federal de Minas Gerais, Belo Horizonte 31270-901, Brazil; 2 Department of Community and Preventive Dentistry, School of Dentistry, Universidade Federal de Minas Gerais, Belo Horizonte 31270-901, Brazil

**Keywords:** facial pain, telemedicine, community dentistry, public health dentistry, COVID-19

## Abstract

This study aimed to identify professional factors associated with case resolution without a referral of orofacial pain to secondary health care by Brazilian Primary Health Care (PHC) practitioners who demanded asynchronous teleconsulting, stratified by year, in 2019 and 2020 (the COVID-19 Pandemic burst). A cross-sectional study employed secondary databases from asynchronous teleconsulting Telehealth Brazil Networks from January 2019 to December 2020. The outcome was the dichotomous variable “If referral to secondary care was avoided.” As covariates: sex, healthcare professions, and category of orofacial pain doubts. A negative binomial regression model estimated each covariate’s unadjusted and adjusted PR (95%CI) and p values, stratified for 2019 and 2020. There was a difference in descriptive factors associated with case resolution without a referral from 2019 to 2020. Females prevailed in both years, and the total demand decreased to a third from 2019 to 2020. The rate of resoluteness decreased by 19.1%. In 2019, nurses (PR = 0.69 CI 95% 0.57–0.83) and other professionals (PR = 0.84 CI 95% 0.73–0.97) showed less frequency of case resolution without a referral than did general dentists. In 2020, oral-cavity-related doubts (PR = 1.18 CI 95% 1.06–1.32) and temporomandibular disorders (PR = 1.33 95% 1.15–1.54) surpassed other causes of orofacial pain in case resolution without a referral, and female professionals avoided referrals more frequently than men (PR = 1.24 CI 95% 1.21–1.38). In conclusion, in 2019, oral cavity doubts and the PHC profession influenced the case resolution. Female professionals and oral cavity doubts scored the higher case resolution without a referral for the service in 2020.

## 1. Introduction

The Brazilian National Health System (SUS) is a universal-equity-based public system. The system provides satisfactory health services and privileges primary care in an unequal and complex society [1]. In primary health care (PHC) units, the main objective is the service resolution without an unnecessary referral, in compliance with international statements that underlie the relevant role of primary health care in pursuing integration, comprehensiveness, and social justice in health [2]. This is also the primary resoluteness followed by the Brazilian telehealth program [1]. In addition, telehealth resources can be essential for disseminating knowledge on and elucidating orofacial pain issues in PHC settings. However, little is known about what determines the resolutive capacity of PHC concerning orofacial pain and TMD issues.

Telehealth uses information technology to enhance health care in distant locations. Due to its low cost and functional characteristics, telehealth can lower the inequalities in health services, reaching poorer groups within an adequate time [3]. Telehealth technology, a term that expands the scope beyond the medical area, represents an important tool available to primary care professionals, solving their doubts and increasing the service’s resoluteness. This resource is paramount, especially in a country with a continental dimension and a heterogeneous health infrastructure distribution, as is the case in Brazil [1]. Expanded to the entire Brazilian territory, covering all five great Brazilian regions (North, Northeast, Midwest, South, and Southeast), the telehealth initiative of the Brazilian Ministry of Health had its activity guidelines defined in 2015 [4]. One of the Program’s strategies is teleconsulting, which consists of bidirectional communication between PHC professionals and teleconsultants (experts in a specific area) for assistance or advanced information on clinical care, health promotion actions, or work process. Teleconsulting is offered by telehealth centers and takes place via synchronous messaging, videoconferences, or asynchronous messages that must be answered within 72 h [5,6]. The primary program goal is to support PHC professionals by offering relevant second opinions. It delivers quick and valuable answers to their questions. This feature enabled a 45% reduction in referrals in some country regions through teleconsulting actions [7].

By contrast, orofacial pain, a broad term encompassing symptoms in the head and neck region, is a frequent form of pain perceived in the face and oral cavity. It may be caused by diseases or disorders of regional structures, nervous system dysfunction, or pain stemming from distant sources [8]. The temporomandibular disorder (TMD), in which painful presentation is a subgroup of orofacial pain, is recognized as a condition of pain or musculoskeletal dysfunction that affects the face in its masticatory structures and encompasses a group of changes involving the temporomandibular joints (TMJ) [9]. It is registered as the primary cause of non-dental pain in the orofacial region and is its most prevalent chronic pain [10]. TMD is defined worldwide as a public health problem [11] in a matrix of multiple possible etiologic factors and interdisciplinary demands [12]. Given its prevalence and relevance in dental practice, knowledge concerning current orofacial pain and temporomandibular disorders in public health services and undergraduate or graduate programs is being debated worldwide [13].

Furthermore, previous evaluation studies with different outcomes have shown that well-structured human resources and management factors have been associated with better performance in Brazilian PHC [14,15]. These topics underline the importance of good health policy initiatives to improve human resources and management in qualified primary care. Hence, spreading and implementing the orofacial pain service in private or public health systems can improve dental practice, providing relief for a series of conditions and avoiding iatrogenic actions or incorrect references.

Therefore, assessing variables of telehealth demands and resolution figures available from the year before the outbreak of the COVID-19 Pandemic and the dissemination of the disease in 2020 is one way to measure and analyze its advantages, shortcomings, and trends over a critical public health period. Accordingly, this study investigated professional factors associated with case resolution without a referral of orofacial pain to secondary health care by Brazilian Primary Health Care (PHC) practitioners who demanded asynchronous teleconsulting, the service dedicated to solving PHC professionals’ doubts about diagnoses issues or work processes, stratified by year, in 2019 and 2020 (the COVID-19 Pandemic burst).

## 2. Materials and Methods

The study used secondary databases from the asynchronous teleconsulting Telehealth Brazil Networks Program from January 2019 to December 2020. The data source was the national database of the Telehealth Results Monitoring and Evaluation System (SMART, the acronym in Portuguese), developed in 2014, provided by the Telehealth Centers that are part of the Telehealth Brazil Networks Program [16]. The telehealth centers were implemented in public universities in 25 out of 26 states in the five Brazilian regions [17]. Duplicate data, incomplete information, or data covering issues other than orofacial pain were excluded. The appropriate University Research Ethics Committee provided ethics approval.

The dichotomous variable “If referral to secondary care was avoided” was the outcome, representing the resolvability of the teleconsulting program. Sex, PHC professional category, and doubts related to orofacial pain were the covariates. Sex was dichotomized in males and females. The categories of PHC practitioners were divided into six groups, according to their relationship with orofacial pain treatment [18] and frequency of appearance in the database, as follows: General Dentists, Specialized Dentists, General Physicians, Specialized Physicians, Nurses, and Others. The “others” embraces administrative staff, auditor-dentists, dental assistants, community health agents, radiology technicians, biomedical, resident physicians, speech therapists, clinical psychologists, physical therapists, pharmacists, occupational therapists, or uninformed.

SMART registered teleconsulting data according to the International Classification of Diseases 10 Version: 2019 (ICD-10) [19] and the International Classification of Primary Care, second edition (ICPC-2) [20]. The last one deals with the reasons for demands beyond the apparent diseases, allowing a better understanding of PHC user problems and perceptions. It is a complementary tool to the traditional ICD and has been gradually recognized as an appropriate classification for family medicine and primary care [21].

The screening of orofacial pain/TMD doubts was based on the American Academy of Orofacial Pain criteria for this study’s purposes [22]. After that, the category of doubts gave rise to three groups based on the proximity to the traditional clinical dental practice, coherent with a current orofacial pain international classification (ICOP) [23] highlighting oral cavity-related pain conditions and temporomandibular disorders. Apart from then, a group for “other conditions in the head and neck” represented the demands that, although referring to the structure of the head/neck, are generally related to other distinct medical specialties, such as headaches and sinusitis, and may hinder or overlap in the oral cavity-related pain or TMD diagnosis.

**Figure 1:**
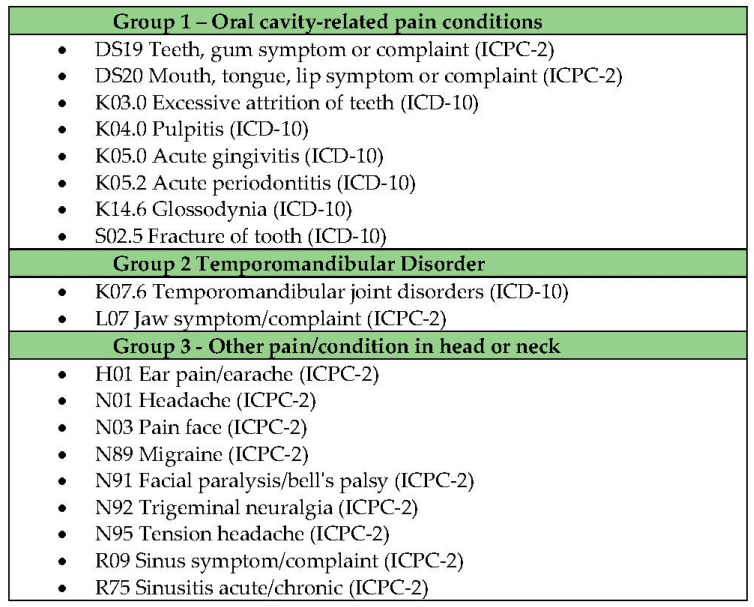
Description of groups of demands/doubts for orofacial pain.

The three demand groups are described in Figure 1.

A descriptive analysis of the data was carried out, using frequency, with data stratification by year of demand (2019 or 2020), for sex and category of the primary care professional, and demands/doubts for orofacial pain. The regression models estimated the prevalence ratios (PR) and the corresponding 95% confidence interval. Initially, it uses unadjusted and adjusted negative binomial regression models to estimate PR (95%CI) and p values for each of the three covariates. Any covariate with a p-value less than 0.25 was a candidate to be tested in the final negative binomial regression model. Only covariates with a p-value less than 0.05 were maintained in the final model [24]. The final model fit was evaluated using a ratio between the residual deviation and the degree of freedom and the chi-square test of the results of the residual deviation. All analyzes were performed in SPSS version 22.0 (SPSS, Chicago, IL, USA).

## 3. Results

**Figure 2:**
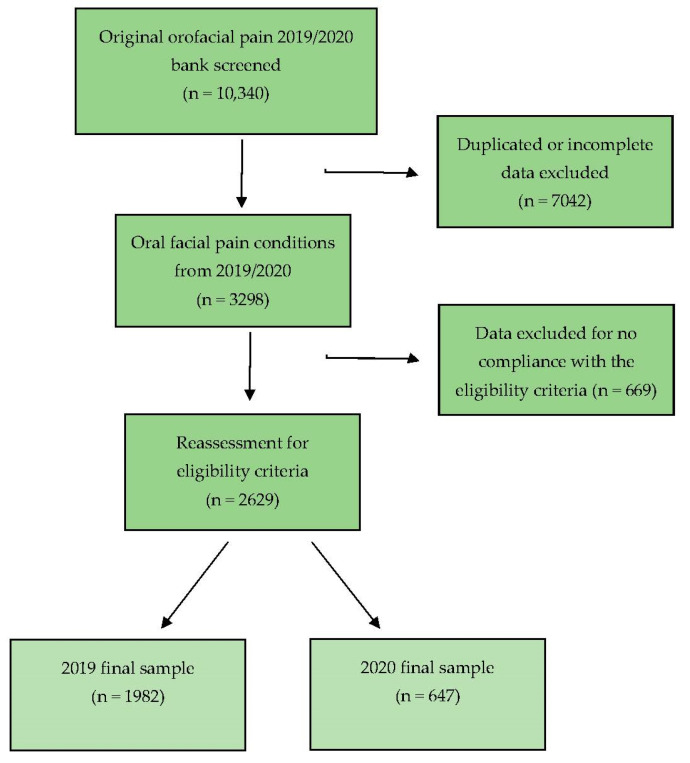
Flow chart showing the criteria of PHC doubts‘ search and selection.

From 10,340 orofacial pain teleconsulting stemming from the original 2019/2020 bank data, 7042 were duplicated or incomplete and excluded. The remaining 3298 were reassessed for compliance with the eligibility criteria, and 669 were discarded. Finally, 2629 integrated the analysis: 1982 referring to 2019 (75.4%) and 647 to 2020 (24.6%) (Figure 2).

From these last, in 2019, 1522 (76.8%) avoided referral to secondary care, and in 2020, 373 (57.7%) did so, representing a reduction of 19.1%. For 2020, 403 (62.3%) PHC professionals were females, increasing the prior frequency of 55.3% for women. Physicians were the most frequent professional category in 2019, and dentists in 2020. Regarding doubts recorded in teleconsulting (ICD/ICPC), the “other” group was the most frequent in 2019 (67.1%), and the oral cavity-related pain conditions group in 2020 (65.5%). Despite the relative growth, general physicians, nurses, “others,” and the G2 (TMD) diagnostic category still represented a minor fraction in 2020 (Table 1).

In 2019, nurses (PR = 0.69 CI 95% 0.57–0.83) and “other professionals” (PR = 0.84 CI 95% 0.73–0.97) showed less frequency of avoiding referral of orofacial pain cases to secondary healthcare than general dentists. When the doubts were related to oral cavity pain conditions (G1), there was a lower frequency of avoiding referral (PR = 0.85 CI 95% 0.77–0.94) than other causes of orofacial pain (G3). In 2020, female professionals avoided referrals more frequently than men (PR = 1.24 CI 95% 1.12–1.38). Oral cavity-related pain conditions (G1) doubts (PR = 1.18 CI 95% 1.06–1.32) and temporomandibular disorders (G2) (PR = 1.33 CI 95% 1.15–1.54) surpassed referral avoidance to secondary care than other cases of orofacial pain (G3) (Table 2).

## 4. Discussion

A change was observed in both the descriptive characteristics of asynchronous teleconsulting on orofacial pain and the factors associated with avoiding referrals to secondary health care from 2019 to 2020. In 2019 (before the COVID-19 Pandemic), physicians were the most frequent professionals demanding teleconsulting for orofacial pain. The majority of the demands were related to non-tooth conditions. In 2020, the first year of the COVID-19 Pandemic, dentists were the most frequent professionals, and oral-cavity-related pain conditions doubts were the most frequent. Noteworthy, Teleconsulting’s ability to avoid referral to secondary health care and the number of demands decreased from 2019 to 2020. In 2019, professional groups and doubts were associated with avoiding referrals. In 2020, the sex of professionals and doubts were associated with this outcome.

The drop in the total teleconsulting over the assessed period stands out. It may represent a consequence of the general global disarray or disruption in the health system caused by the Pandemic, which forced organizations to focus on COVID-19 issues rather than regular services. Services were suddenly rearranged to deal mainly with pandemic issues or urgencies, leaving behind some previously structured programs and other essential health needs. In Brazil, mobility and non-essential services suffered severe restrictions in 2020, particularly from the second trimester, following the worldwide spread of cases and deaths [25]. Similarly, the apparent drop in physicians’ demands in 2020 coincides with the burden of the COVID-19 Pandemic. The mobility restrictions prevented in-person health facility visits and face-to-face patient–doctor interaction, and the shift from traditional care to telehealth occurred for a limited period, demanding rapid training and personnel allocation [26]. In this scenario, the physicians may have initially interrupted regular and elective procedures in favor of medical urgencies, not to mention the staff directly involved with COVID-19 patient management. This fast and somewhat chaotic change may explain the withdrawal of physicians in teleconsulting devoted to orofacial pain issues in the stressful pandemic context of 2020 [27]. Concerning types of doubts, the preeminence of the G3 group over G1 and G2 in 2019 and the reverse in 2020 follows this same scenario, as “other conditions in the head and neck” represented the demands in general related to medical specialties, such as headaches and sinusitis.

The dentists themselves and the doubt categories related to oral cavity conditions (G1) were the most frequent in the 2020 sample. It matches the reallocation of dental professionals in the Public Health System during the Pandemic, leaving their previous routine in favor of managing face-to-face dental urgencies, potentially leading several dental branches to search for information on acute dental conditions in teleconsulting. It is important to note that dental pain is reported as a relevant fraction of dental urgencies [28] and represents the most frequent category of orofacial pain [29]. The general 2020 increase in females in the sample matches the increase in dentists. Women also represent a relevant fraction of Brazilian dental schools in the national dental public health system [30]. In contrast, the G2 (TMD) diagnostic category represented a minor fraction in both years assessed. This situation may reflect the mechanical and technical classical tendencies of dental formation, contrasting with the complexity of chronic conditions such as TMD, which tend to be overlooked in favor of the relative simplicity of acute urgent pathologies. The novelty of the TMD/orofacial pain field in dental schools may also contribute [31].

In 2019 (the pre-pandemic year), nurses and other professionals showed a lower resolution without a referral performance than general dentists. Despite the notorious wide range of diagnostic conditions involved and interdisciplinarity, orofacial pain is traditionally a dental branch that was gradually recognized as a dental specialty. In Brazil, the area has been considered a separate dental specialty from the Federal Council of Dentistry rule since 2002 [31]. This specific dental background in orofacial issues would give dentists a higher capacity for resoluteness in this field than nurses and other professionals. By contrast, in 2019, the lower frequency of case resolution without a referral from oral cavity issues could be partly explained by the full availability of the secondary service chain. In an average period, without pandemic restrictions, the steady health system flow to secondary aid permits PHC professionals to refer a higher number of mild or moderate cases. This standard would change during the Pandemic.

The upsurge in the Pandemic in 2020 marks the preeminence of dentists in the sample. Nurses and “other professionals” in 2020 did not show the same negative association as in 2019. This situation may well reflect the staff reallocation and training in a disruptive period to deal with acute dental concerns, granting fewer referrals (some “other professionals” were already dental practice-related, such as dental assistants). The increase in the proportion of dentists in the sample could result in more resolution capacity of G1 and G2 doubts [31]. Women’s higher performance may also be associated with their higher commitment to health care during a pandemic, requiring more in-depth investigation [32,33].

The severe acute respiratory syndrome caused by Coronavirus 2 (SARS-COVID 2), or COVID-19, with the surge in 2020, still represents a massive problem to healthcare systems worldwide, with millions of dead by that year. A “Post-Covid Syndrome” can last beyond the acute 4-week period and affects multiple organs and systems, also related to widespread pain (myalgia) and headaches [34]. Although still under debate and extensive investigation, it will inevitably require interdisciplinary health teams for its study, control, and surveillance, most likely for extended periods. In this regard, telehealth (encompassing teleconsulting) for managing chronic conditions must also find a fertile field of application and expansion ahead [35,36]. Notwithstanding the eventual distortions, challenges in implementation, and lack of randomized controlled assessments of its clinical outcomes and long-term economic analyses [37] these technological advantages are paramount to reducing inequities in periods of high health challenging demands, much like the outbreak of the COVID-19 Pandemic in 2019–2020 [38].

PHC is structured to offer solutions for the basic needs of health, reducing the number of demands for secondary services, mitigating costs, and making the whole system more efficient. The drop in resoluteness recorded in the period of this research following the Pandemic challenges may also reflect a repressed demand for health, making simpler pathologies develop and escalate to a matrix of more complex conditions over the same period [25,27]. It would naturally deflagrate secondary actions, with the potential to decrease the total resoluteness aspect.

This study presents some limitations. First, the short period covering data investigation (2019 and 2020) may not reflect the impact of previous or posterior tendencies upon the Telehealth usage characteristics; therefore, comprehensive time-covering data analysis still needs to be conducted. Second, the cross-sectional study design does not enable inferences regarding causality. Third, the effect of other covariates, such as professional age and patient characteristics on demographic (i.e., age, gender) and clinical status (i.e., the severity of pain and its length and quality, systemic background of the patients) were not available in this dataset, so the study considered only some PHC personnel’s dataset. It is important to suggest to the Brazilian Ministry of Health the inclusion of both age of the patient and the professional, a truly confounding variable in quantitative studies [39]. Moreover, more details in the clinical diagnosis of the patient may be also very useful to understand factors associated with our outcome. The access to these variables could impact the quality of the associations identified. Despite these limitations, the study contributes to future analyses regarding the Brazilian orofacial pain teleconsulting program and to elaborate a historical time series research.

## 5. Conclusions

In 2019, oral cavity doubts scored the lower-case resolution without a referral and PHC profession also influenced this outcome. Female professionals and oral cavity doubts scored the higher case resolution without a referral for the service in 2020.

## Figures and Tables

**Table 1:** Description of the characteristics of telehealth for orofacial pain in the Unified Health System, Brazil, 2019 and 2020.

Variable	2019 (N = 1982) Frequency N (%)	2020 (N = 647) Frequency N (%)
If avoided the referral to secondary care		
No	460 (23.2)	274 (42.3)
Yes	1522 (76.8)	373 (57.7)
Sex of PHC * professional		
Female	1096 (55.3)	403 (62.3)
Male	886 (44.7)	244 (37.7)
PHC * profession		
General dentist	171 (8.6)	149 (23.0)
Specialized dentist	332 (16.8)	220 (34.0)
General physician	16 (0.8)	14 (2.2)
Specialized physician	1284 (64.8)	135 (20.9)
Nurse	91 (4.6)	46 (7.1)
Others	88 (4.4)	83 (12.8)
Demands/Doubts		
Group 1	619 (31.2)	424 (65.5)
Group 2	33 (1.7)	27 (4.2)
Group 3	1330 (67.1)	196 (30.3)

* Primary Health Care.

**Table 2:** Factors associated with avoiding orofacial pain referral to secondary healthcare in the Unified Health System, Brazil, 2019 and 2020 telehealth.

Variables	2019				2020			
	Unadjusted PR (CI 95%)	*p*-Value	Adjusted PR (CI 95%)	*p*-value	Unadjusted PR (CI 95%)	*p*-Value	Adjusted PR (CI 95%)	*p*-Value
**Sex of PHC** *								
Female	0.97 (0.94–0.99)	0.015			1.27 (1.14–1.41)	<0.001	1.24 (1.12–1.38)	<0.001
Male	1				1		1	
**PHC** * profession								
Others	0.88 (0.76–1.01)	0.065	0.84 (0.73–0.97)	0.019	0.90 (0.78–1.04)	0.168		
Nurse	0.75 (0.63–0.89)	0.001	0.69 (0.57–0.83)	<0.001	0.89 (0.74–1.08)	0.232		
Specialized Physician	1.15 (1.08–1.22)	<0.001	0.99 (0.88–1.11)	0.828	0.82 (0.72–0.95)	0.006		
General Physician	0.96 (0.75–1.22)	0.719	0.82 (0.64–1.07)	0.129	0.99 (0.77–1.27)	0.952		
Specialized Dentist	0.96 (0.89–1.04)	0.330	0.96 (0.89–1.04)	0.354	0.95 (0.86–1.05)	0.275		
General Dentist	1		1		1			
**Doubts**								
Group 1	0.82 (0.79–0.86)	<0.001	0.85 (0.77–0.94)	0.001	1.21 (1.08–1.36)	0.001	1.18 (1.06–1.32)	0.040
Group 2	0.92 (0.81–1.04)	0.175	0.96 (0.82–1.11)	0.551	1.39 (1.19–1.62)	<0.001	1.33 (1.15–1.54)	0.001
Group 3	1		1		1		1	

* Primary Health Care.

## Data Availability

The data presented in this study are available on request from the corresponding author.
